# 中国易瑞沙扩大用药项目中晚期非小细胞肺癌长期生存患者的研究

**DOI:** 10.3779/j.issn.1009-3419.2012.06.03

**Published:** 2012-06-20

**Authors:** 龙芸 李, 巍 钟, 美琳 廖, 黎 陈, 宝惠 韩, 忠震 管, 世英 于, 叙仪 刘, 一龙 吴, 国梁 蒋, 建明 徐, 嘉 陈, 敏 陶, 荣城 罗, 为民 李, 农 徐, 肖 赵, 孟昭 王

**Affiliations:** 1 100730 北京，中国医学科学院北京协和医学院北京协和医院 Department of Respiratory Diseases, Peking Union Medical College Hospital, Peking Union Medical College and Chinese Academy of Medical Sciences, 100730 Beijing, China; 2 200030 上海，上海交通大学附属胸科医院 Lung Tumor Clinical Medicine Center, Shanghai Chest Hospital, Shanghai Jiaotong University, 200030 Shanghai, China; 3 250117 济南，山东省肿瘤医院 Department of Oncology, Shandong Cancer Hospital, 250117 Jinan, China; 4 510060 广州，中山大学附属肿瘤医院 Department of Medical Oncology, Sun Yat-Sen University Cancer Center, 510060 Guangzhou, China; 5 430030 武汉，华中科技大学同济医学院附属同济医院 Cancer Center, Tongji Hospital, Tongji Medical College, Huazhong University of Science and Technology, 430030 Wuhan, China; 6 100142 北京，北京肿瘤医院 Department of Thoracic Medical Oncology, Peking University School of Oncology, Beijing Cancer Hospital and Institute, 100142 Beijing, China; 7 510080 广州，广东省人民医院 Guangdong Institute and Cancer Center, Guangdong General Hospital, 510080 Guangzhou, China; 8 200032 上海，复旦大学附属肿瘤医院 Departments of Radiation Oncology, Fudan University Shanghai Cancer Center, 200032 Shanghai, China; 9 100071 北京，中国人民解放军307医院 Department of Oncology, Affiliated Hospital of Academy of Military Medical Sciences, 100071 Beijing, China; 10 210009 南京，江苏省肿瘤医院 Department of Oncology, Jiangsu Cancer Hospital, 210009 Nanjing, China; 11 215006 苏州，苏州大学第一附属医院 Department of Oncology, the First Affiliated Hospital of Soochow University, 215006 Suzhou, China; 12 510515 广州，南方医院 Cancer Center, Nanfang Medical University Nanfang Hospital, 510515 Guangzhou, China; 13 610041 成都，四川大学华西医院 Department of Respiratory Diseases, West China Hospital, West China School of Medicine Sichuan University, 610041 Chengdu, China; 14 10003 杭州，浙江大学医学院附属第一医院 Department of Chemotherapy, the First Affiliated Hospital of Medical School of Zhejiang University, 310003 Hangzhou, China

**Keywords:** 肺肿瘤, 吉非替尼, 中国易瑞沙扩大用药, 长期生存, Lung neoplasms, Gefitinib, Expand Access Program (EAP), Long-term survival

## Abstract

**背景与目的:**

中国易瑞沙（吉非替尼、ZD1839）扩大用药（EAP）项目开始于2001年，旨在为标准治疗失败后或不能耐受化疗的晚期非小细胞肺癌（non-small cell lung cancer, NSCLC）患者提供吉非替尼单药治疗。本研究主要评估仍在EAP组长期生存者的生活质量、肿瘤控制情况、服药安全性、临床特点及基因测定。次要目标为探讨长期生存者的临床特征。

**方法:**

这是一项描述性观察研究，数据收集依据流行病学研究方法。对于仍在EAP组中的患者，数据将采用横断面调查的方法；对于已从EAP组中退出的长期生存者和快速进展者，数据以回顾性方式收集。

**结果:**

在EAP数据库中共筛选出符合条件的患者934例，其中59例为服用吉非替尼> 3年的长期生存者，包括25例仍在EAP组，34例已退出EAP组，875例为快速进展者。59例长期生存者来自中国的15个临床中心。仍在EAP组的长期生存者，中位肺癌治疗功能评价量表（functional assessment of cancer therapy-lung, FACT-L）、试验结果系数（trial outcome index, TOI）和肺癌量表（lung cancer subscale, LCS）分值分别为64.5、37和12.5，91.6%患者的体力状态（performance status, PS）为0分-1分，客观有效率、疾病控制率和中位有效持续时间分别为37.5%、87.5%和68个月。在59例长期生存者中，没有发现严重或新的不良反应；其中 < 65岁者68.5%，腺癌81.4%，女性55.9%，从不吸烟者71%。与快速进展者比较，长期生存者中女性比例稍高（*P*=0.02）。本研究收集了3例患者的组织标本，其中1例*EGFR*突变阳性；2例Ki-67蛋白表达阳性，这2例患者生存期都超过73个月。同时收集了22例血浆标本，1例*EGFR*突变阳性。

**结论:**

部分晚期NSCLC应用吉非替尼治疗可获3年以上长期生存，并为长期生存者提供了良好的生活质量、满意的疗效和药物耐受性。

## Abstract (en)

**Background and objective:**

The Expand Access Program (EAP) of Iressa(gefitinib, ZD1839) in China was initiated in 2001 with the aim of providing gefitinib to non-small cell lung cancer (NSCLC) patients who failed to respond to standard treatment or who could not tolerate chemotherapy. The primary objective was to describe the quality of life (QoL), tumor control status, drug safety, and clinical/genomic features of active long-term survivors enrolled in the EAP. The secondary objective was to determine the clinical characteristics of long-term survivors in the EAP program.

**Methods:**

In this descriptive observational study, data were collected based on epidemiological research methods. The data of patients who were actively participating in the EAP and still undergoing gefitinib treatment were collected in a cross-sectional manner to reflect the current status of each patient. Meanwhile, the data of patients who had been on gefitinib treatment for more than three years and had already been terminated from the EAP or those who were fast progressors were collected retrospectively.

**Results:**

A total of 934 patients were screened in the EAP database. Among these patients, 25 were active long-term survivors still enrolled in the EAP and 34 were long-term survivors who had been terminated from the program. These 59 patients were enrolled in 15 different centers in China, and the remaining 875 patients were fast progressors. The median scores for the Functional Assessment of Cancer Therapy-Lung (FACT-L), Trial Outcome Index (TOI), and Lung Cancer Subscale (LCS) of the 25 long-term survivors were 64.5, 37 and 12.5, respectively. The performance status 0-1 accounted for 91.6% of the data observed during the cross-sectional survey. For active long-term survivors, the objective response rate was 37.5%, the disease control rate was 87.5%, and the median duration of response time was almost 68 months. In the long-term survivor group, no serious and new adverse events were reported. Patients who were aged under 65 years (68.5%), affected with adenocarcinoma (81.4%), female (55.9%), or had never smoked (71%) accounted for majority of the long-term survivors. The percentage of females was significantly higher in the long-term survivor group than in the fast progressor group (*P*=0.02). Three tissue samples were collected from each of the 24 active long-term survivors, and one patient was found to be positive of *EGFR* mutation. Twenty-two blood samples were also collected, and one patient tested positive for *EGFR* mutation. The Ki67 protein expression was also tested in three tissue samples, and two of these were found positive for Ki67 protein expression, with a response duration time of over 73 months.

**Conclusion:**

A 250 mg dose of gefitinib offers good QoL and is safe for advanced NSCLC long-term survivors even after more than three years of treatment. According to the evaluation of the current tumor control statuses of patients, gefitinib demonstrates good efficacy in these active long-term survivors.

易瑞沙（吉非替尼、ZD1839）扩大用药项目（Expand Access Program, EAP 1839IL/0052）于2001年-2007年在中国进行，旨在为标准治疗失败、无法接受其它系统性抗肿瘤治疗、无法耐受化疗、或因无法或不适用其它吉非替尼临床研究的非小细胞肺癌（non-small cell lung cancer, NSCLC）患者提供吉非替尼单药治疗，入选该项目患者约1, 600例。根据EAP方案，只要医生判定患者能从该治疗中受益，该患者将一直免费获得吉非替尼治疗。

文献^[1-4]^已报道部分NSCLC患者能长期从吉非替尼治疗受益，这部分患者的临床特征包括女性、非鳞癌、不吸烟和肿瘤组织有表皮生长因子受体（epidermal growth factor receptor, EGFR）基因敏感性突变等。本EAP项目中为数不少（76例）患者服用吉非替尼已超过3年。因此，评估EAP长期生存者的生活质量、肿瘤控制情况、安全性和临床/基因组特征具有一定的临床价值，以便更好指导临床治疗。

## 1 研究目的、方法与对象

本研究采用对EAP患者描述性、观察性的横断面调查和回顾性分析，无预先设定的统计假设，不干预EAP组中患者当前的治疗。研究获得了伦理委员会的审核及批准，对于仍在EAP组的长期生存者均需获得知情同意书。服药超过3年已退出EAP组的长期生存者和快速进展者仅作回顾性分析，因此免除知情同意书。

### 1.1 研究目的

#### 1.1.1 主要目标

通过横断面调查评估仍在EAP组的长期生存者的生活质量、临床特点、疗效及用药安全性。

#### 1.1.2 次要目标

对服药超过3年已退出EAP组的长期生存者及快速进展者进行回顾性资料收集，以便行有关预后风险因素分析。

#### 1.1.3 探索性目标

评估仍在EAP项目中长期生存者的肿瘤组织和外周血标本*EGFR*基因突变情况，并测定肿瘤组织中Ki-67蛋白表达与生存的关系。

### 1.2 研究步骤、设计和流程

见图 1。

**1:**
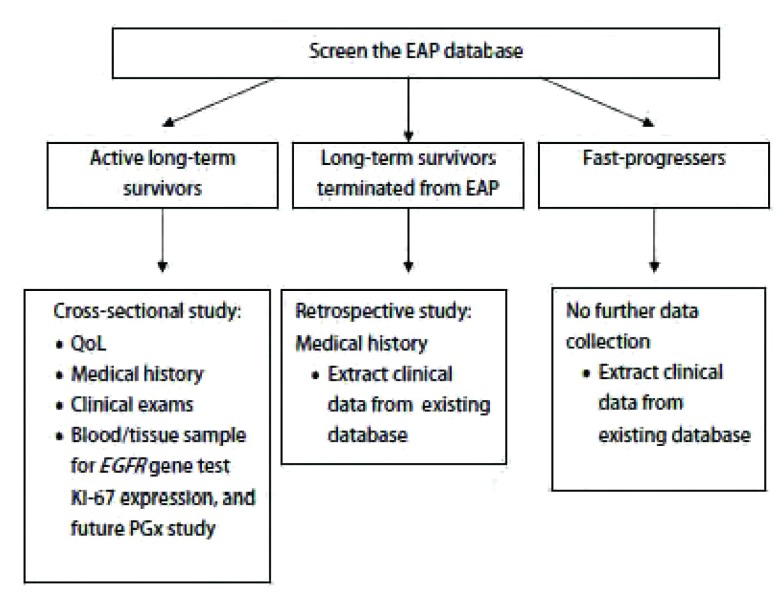
总试验设计和流程 Flow chart of study design

### 1.3 研究对象的入组标准

#### 1.3.1 入选横断面研究的对象标准

入选的研究对象必须符合以下标准：流程开始前需签署知情同意书；已确诊为NSCLC；参加中国EAP已超过3年，且至今仍在EAP组服用吉非替尼；患者同意提供血标本和存档肿瘤组织标本进行*EGFR*基因和Ki-67蛋白检查。

#### 1.3.2 入选回顾性研究的对象标准

入选的研究对象必须符合以下标准：没有对使用现有医疗数据作为科学研究的书面反对意见；参加了中国易瑞沙EAP项目；组织学或细胞学确诊患有NSCLC；已退出EAP组的长期生存者必须从EAP项目中连续接受吉非替尼治疗超过3年；快速进展者为自获得EAP赠药后，第1次随访时因疾病已进展而出组。

### 1.4 仍在EAP组长期生存患者的研究变量的收集

#### 1.4.1 资料收集

收集临床资料，并在相关的病例报告表中记录，包括出生日期、性别；WHO体力状况（performance status, PS）（进入EAP前）；病史，包括病理组织学、分期和入组EAP前的抗肿瘤治疗；入组EAP前的吸烟史；开始吉非替尼治疗时的分期和PS；入组EAP后的合并抗肿瘤治疗。记录CT/MRI扫描结果，按RECIST（Response Evaluation Criteria in Solid Tumors）标准评估目前肿瘤控制状况；评估入组患者的吉非替尼治疗依从性；记录不良事件（adverse event, AE）和严重不良事件（serious adverse event, SAE）。

每位患者应独立填写癌症治疗功能评价量表（functional assessment of cancer therapy-lung, FACT-L）。如果患者无法自行完成问卷，中心研究者可以向患者阅读问题，但不能试图影响患者给予的答案。在完成FACT-L问卷后，研究人员必须立即检查问卷完成情况，如有遗漏，应将问卷还给患者，立即让患者补充。并计算出所有问卷FACT-L总分、试验结果系数（trial outcome index, TOI）和肺癌量表（lung cancer subscale, LCS）的中位值和范围。

每例患者应进行体格检查，包括身高、体重、体温、血压和呼吸系统检查；血液常规、血液生化和尿液测定。

吉非替尼治疗的顺应性由下列公式得出：

$
顺应性 = \frac{{\left( {{\rm{the}}\;{\rm{times}}\;{\rm{of}}\;{\rm{gifitinib}}\;{\rm{pills}}\;{\rm{distributed-1}}} \right) \times 100}}{{\left( {{\rm{the}}\;{\rm{date}}\;{\rm{of}}\;{\rm{the}}\;{\rm{last}}\;{\rm{distribution-the}}\;{\rm{date}}\;{\rm{of}}\;{\rm{the}}\;{\rm{first}}\;{\rm{distribution}}} \right)}}
$

#### 1.4.2 访视窗

对于仍在EAP组的患者，考虑到现实中对晚期癌症患者的常规诊疗措施，可在访视第1天后的1周内完成临床检查。因本研究是横断面研究，除了对与治疗相关的严重不良事件处理及随访外，未计划再对患者进行随访。

#### 1.4.3 生物标记物分析

本次研究中使用蝎型探针扩增阻滞突变系统（scorpions amplification refractory mutation system, ARMS）法和直接测序法对存档的肿瘤标本和外周血游离DNA检测*EGFR*基因突变。应用免疫组化判断组织中Ki-67蛋白表达。

### 1.5 已出组的长期生存者和快速进展者的资料获取

对已出组的长期生存者和快速进展者可以从资料库回顾性得到关键临床资料。

### 1.6 统计分析方法

使用描述性统计方法总结本次研究的生存质量（quality of life, QoL）、疗效、安全性、治疗依从性和横断面调查时长期生存者的实验室分析结果。对长期生存者和快速进展者的关键临床特征比较行卡方检验。根据临床资料行可能风险描述。*P* < 0.05为差异有统计学意义。

## 2 结果

### 2.1 患者构成

患者来自中国的15个研究中心。在EAP数据库中共筛选出符合条件的患者934例，服用吉非替尼超过3年的长期生存者59例，其中仍在EAP组的长期生存者25例，已退出EAP组的长期生存者34例，快速进展者875例。

### 2.2 长期生存者的人口学和临床特征

吉非替尼治疗超过3年的长期生存者，中位年龄56.6岁，≥65岁的老年人31.5%，男性、女性分别为44.1%和55.9%。既往接受化疗者89.7%、手术57.6%、放疗48.3%，腺癌81.4%，Ⅲ期/Ⅳ期患者94.6%，从不吸烟者70.7%。接受易瑞沙治疗的长期生存者依从性较好，94.9%的患者依从率在80%-100%之间（表 1）。

**1:** 长期生存者人口学和临床特征 Demography and clinical characteristics of long-term survivors

	Active long-term survivor (*n*=25)	Long-term survivor terminated from EAP (*n*=34)	Total (*n*=59)
Age (year)			
Median	58.4	52.45	56.6
Missing	1	4	5
Age distribution [n (%)]			
< 65 years	15 (62.50)	22 (73.33)	37 (68.52)
≥65 years	9 (37.50)	8 (26.67)	17 (31.48)
Missing	1	4	5
Sex, n(%)			
Fem ale	14 (56.00)	19 (55.88)	33 (55.93)
Male	11 (44.00)	15 (44.12)	26 (44.07)
Pathology [n (%)]			
Squamous carcinoma	3 (12.00)	1 (2.94)	4 (6.78)
Adenocarcinoma	17 (68.00)	31 (91.18)	48 (81.36)
Bronchiolo alveolar carcinoma	4 (16.00)	2 (5.88)	6 (10.17)
Others	1 (4.00)	0	1(1.69)
Smoking history [n (%)]			
Never smoker	17 (68.00)	24 (72.73)	41(70.69)
Ever smoker	8 (32.00)	8 (24.24)	16(27.59)
Smoker	0	1 (3.03)	1(1.72)
Missing	0	1	1
Stage [n (%)]			
Ⅰ, Ⅱ	2 (8.00)	1 (3.33)	3(5.45)
Ⅲ, Ⅳ	23 (92.00)	29 (96.67)	52 (94.55)
Missing	0	4	4
Previous treatment [n (%)]			
No	0	4 (11.76)	4 (6.78)
Yes	25 (100.00)	30 (88.24)	55 (93.22)
Previous operation [n (%)]			
No	9 (36.00)	16 (47.06)	25 (42.37)
Yes	16 (64.00)	18 (52.94)	34 (57.63)
Previous chemotherapy [n (%)]			
No	2 (8.00)	4(12.12)	6 (10.34)
Yes	23 (92.00)	29 (87.88)	52 (89.66)
Missing	0	1	1
Previous radiotherapy [n (%)]			
No	12 (48.00)	18 (54.55)	30 (51.72)
Yes	13 (52.00)	15 (45.45)	28 (48.28)
Missing	0	1	1
Compliance			
Median	0.97	1	1
Compliance [n (%)]			
> 120	0	0	0
80-120	24 (96.00)	32 (94.12)	56 (94.92)
< 80	1 (4.00)	2 (5.88)	3 (5.08)
Chemotherapy in EAP [n (%)]			
No	22 (88.00)	22 (68.75)	44 (77.19)
Yes	3 (12.00)	10 (31.25)	13 (22.81)
Missing	0	2	2
Radiotherapy in EAP [n (%)]			
No	23 (95.83)	24 (75.00)	47 (83.93)
Yes	1 (4.17)	8 (25.00)	9 (16.07)
Missing	1	2	3
Targeted therapy (only taken gefitinib in EAP) [n (%)]			
No	24 (96.00)	27(87.10)	51 (91.07)
Yes	1 (4.00)	4(12.90)	5 (8.93)
Missing	0	3	3
Treatment in EAP [n (%)]			
No	22 (88.00)	19 (59.38)	41 (71.93)
Yes	3 (12.00)	13 (40.62)	16 (28.07)
Missing	0	2	2

### 2.3 仍在EAP组的长期生存患者生活质量、疗效及安全性评价

见表 2。

**2:** 仍在EAP组长期生存患者生活质量及疗效 Quality of life and efficacy of the active long-term survivors

	Active long-term survivor (*n*=25)
FACT-L score	
N (Missing)	24 (1)
Median	64.5
TOI score	
N (Missing)	24 (1)
Median	37
LCS score	
N (Missing)	24 (1)
Median	12.5
Efficacy with RECIST criteria [n (%)]	
CR	6 (25.00)
PR	3 (12.50)
PD	1 (4.17)
SD	12 (50.00)
Objective response rate [n (%)]	9 (37.50)
Disease control rate [n (%)]	21 (87.50)
NE	2 (8.33)
Missing	1
Quartile of the duration of response time (month)	
25%	73.77
50%	67.77
75%	62.53
Performance status [n (%)]	
Normal activity (0)	20 (83.33)
Restricted but ambulatory (1)	2 (8.33)
Up more than 50% of waking hours (2)	1 (4.17)
Confined to bed or chair more than 50% (3)	1 (4.17)
Totally confined to bed (4)	0
Missing	1
CR: complete response; PR: partial response; PD: progressive disease.	

#### 2.3.1 生活质量及肿瘤控制情况

25例仍在EAP组长期生存者，96%完成了FACT-L生活质量问卷，中位FACT-L、TOI及LCS分值分别为64.5、37和12.5。对体能状况（physical well-being, PWB）中“我体力欠缺”问题的回答：70.8%的患者无体力下降，20.8%感到体力稍有下降。功能状况（functional well-being, FWB）中“我能够享受生活”的回答：50%非常享受生活，29.2%有一点享受生活。对“我对现在的生活质量感到满意”的回答：62.5%非常满意，12.5%有些满意。91.6%的患者PS评分为0分-1分。因此吉非替尼持续治疗3年后，大部分患者仍有良好的生活质量。使用RESIST标准评估仍在EAP组的长期生存者的当前肿瘤控制情况，1例出现疾病进展，客观有效率37.5%，疾病控制率87.5%，中位疗效持续时间为67.8个月，证实了吉非替尼的良好疗效。

#### 2.3.2 与预后相关的风险因素

仍在EAP组中的88%患者一直应用吉非替尼治疗，有少数患者因疾病进展行化疗（12%）、放疗（4%）及其它靶向治疗或又重复吉非替尼治疗（4%）。而退出EAP组长期生存者行化疗、放疗及靶向治疗分别为31%、25%及12.9%。

#### 2.3.3 长期生存者与快速进展者的主要临床特征比较

长期生存者与快速进展者的主要临床特征比较，两组患者年龄分布相似。长期生存组中女性患者比例高于快速进展组（55.9% *vs* 40.8%, *P*=0.02）（表 3）。

**3:** 长期生存者和快速进展者主要临床特征 The key clinical features of long-term survivors and fast-progressers

	Long-term survival	Fast-progresser	*P*
Age [n (%)]			0.423, 8
< 65 years	37 (68.52)	577 (73.50)	
≥65 years	17 (31.48)	208 (26.50)	
Total	54	785	
Gender [n (%)]			0.022, 7
Female	33 (55.93)	351 (40.81)	
Male	26 (44.07)	509 (59.19)	
Total	59	860	
Previous treatment [*n* (%)]			0.143, 3
No	4(6.78)	100 (13.42)	
Yes	55(93.22)	645 (86.58)	
Total	59	745	
Disease stage [n (%)]			0.000, 2
Ⅰ, Ⅱ	3 (5.45)	0 (0.00)	
Ⅲ, Ⅳ	52 (94.55)	859 (100.00)	
Total	55	859	

#### 2.3.4 探索性变量

本研究收集到3例组织标本，仅1例*EGFR*基因突变阳性（L858R），该病例为女性，58岁，不吸烟，肺鳞癌，服用吉非替尼疗效持续时间为67.8个月。22例血浆标本中1例*EGFR*基因突变阳性（外显子19缺失），也为女性，不吸烟，晚期肺腺癌。3例组织标本中，有2例Ki67蛋白表达阳性，疗效持续时间均超过73个月。

#### 2.3.5 安全性评估

评估显示经长期吉非替尼治疗患者的安全性与以往文献报道相似。仅1例患者出现2度血小板下降。1度-2度肌酐异常3例（12.5%），≥3度1例（4%）。未出现新的肝酶异常报告，1度-2度ALT（alkaline phosphatase）3例（12.5%），≥3度AST（aspartate aminotransferase）1例（4%）。血钠、血钙≥3度各1例。尿液分析可见轻度异常的血尿5例（22%）。不良事件详见表 4。

**4:** 不良事件列表 Number (%) of patients who had an adverse event in any category

	Long-term survivor terminated from EAP (*n*=34)			Active long-term survivor (*n*=25)		
	Number of subjects	%		Number of subjects	Number of events	%
All adverse events	0	0		1	2	4
Adverse events in relation to research drug	0	0		1	1	4
Serious adverse events	0	0		0	0	0
Adverse events leading to termination	0	0		0	0	0
Adverse events leading to death	0	0		0	0	0

## 3 讨论

EAP项目是易瑞沙扩大用药的慈善项目，项目开始时无统计研究设计。在项目开展过程中显示晚期NSCLC患者治疗明显获益。本研究共收集到长期生存者资料59例，如此长的生存期在化疗时代确为罕见。为了探讨吉非替尼长期治疗的安全性和有效性，因此对仍在EAP组中3年以上长期生存患者的生活质量、疗效、安全性及临床特征等行描述性研究。

### 3.1

横断面调查了仍在EAP组中治疗的患者，治疗结果满意。

#### 3.1.1 QoL和PS

QoL又称生存质量或生命质量，即患者的主观指标，有助于全面、系统地评估肺癌患者生理功能、心理情绪、社会健康状况及治疗效果，为制定个体化的治疗方案提供理论依据，也是目前选择抗肿瘤治疗必须考虑的重要因素^[5]^。

本研究收集的QoL是仍在EAP组治疗的长期生存患者目前状况，但无基线对照。QoL经FACT-L问卷评价，问卷调查的顺应性非常高（96%）。中位FACT-L、TOI、LCS评分分别为64.5、37及12.5。PWB中70.8%的患者感到无体力下降，20.8%感到体力稍有下降。FWB中50%的患者非常享受生活，62.5%对目前生活非常满意。PS是QoL的重要内容之一，用于评价患者当前的功能状况，因此从这个层面也可反映患者总体健康状况。本研究中PS 0分-1分的患者占91.6%。经QoL的评估，显示经过吉非替尼250 mg/d治疗3年以上，患者依然保持良好的生活质量。

#### 3.1.2 肿瘤控制状况

RECIST评价显示，仅1例患者出现疾病进展，客观有效率为37.5%，疾病控制率为87.5%，中位疗效持续时间为67.8个月，以上均显示接受吉非替尼长期治疗者仍能保持良好的疗效，为晚期NSCLC开辟了新的治疗里程。

#### 3.1.3 安全性评估

经吉非替尼治疗的长期生存患者显示了非常好的耐受性。AES与以往报道类似，无严重不良事件和导致停药或死亡的不良事件发生。临床实验室检查结果也同以往报道^[6]^。无新的安全相关事件报道。仅少数有ALT和/或AST及肌酐的异常，均为轻度到中度。血钠、血钙≥3度各1例。轻度血尿异常5例。虽然患者的不良事件分级低，发生例数少，但长期应用吉非替尼治疗者仍应注意定期检查^[7]^。

### 3.2 预后相关风险因素分析

#### 3.2.1 吉非替尼治疗后长期生存的预后相关风险因素

曾报道选择性的NSCLC患者可从吉非替尼治疗中长期受益，这些患者的临床特征包括女性、非鳞癌和不吸烟。本研究入选的患者多数为腺癌经化疗失败者和Ⅲ期/Ⅳ期NSCLC患者^[3]^。其中 < 65岁、女性和不吸烟患者比例高于65岁（含65岁）以上、男性和曾经或正在吸烟者，与以往报道相同。但本研究也显示男性、老年人也可在吉非替尼治疗中有长期生存获益。由于EAP较早在中国开展，治疗失败后伴随其它治疗的比例很低（28%），如化疗、放疗和靶向治疗。需伴随其他抗肿瘤治疗也可能是吉非替尼治疗预后潜在风险因素。但部分患者经有关治疗，再重复吉非替尼治疗，能继续明显延长他们的生存期。本研究中长期生存者PS 0分-1分的患者占91.6%。再次证实PS是吉非替尼治疗的预后因素。

#### 3.2.2 快速进展患者和长期生存患者主要临床特征比较

原期盼快速进展患者和长期生存患者主要临床特征的比较可印证预后风险。由于快速进展患者信息源于EAP资料，仅包括性别、年龄、既往治疗和疾病分期。两组显示年龄、既往治疗及疾病分期百分数相似。仅长期生存患者中女性比例稍高于快速进展者（55.93% *vs* 40.81%, *P*=0.02），与以往报道相似。

### 3.3 生物学标记物探索肿瘤组织和血浆标本*EGFR*基因突变情况

以往研究^[1, 6, 8-10]^显示*EGFR*基因突变是吉非替尼治疗的预测因素。常见的*EGFR*基因敏感突变为外显子19的缺失突变和外显子21的点突变（L858R）。具有*EGFR*基因敏感突变的患者，吉非替尼治疗的效果显著，无疾病进展时间（progression free survival, PFS）9.2个-10.8个月，总生存时间达30.5个月。外周血循环DNA检测*EGFR*基因突变的尝试使其更有临床意义。本研究中25例仍在EAP组长期生存患者中，24例提供组织或血浆标本行探索性研究，收集到3例组织标本，有1例L858R突变，22例血浆标本中1例外显子19缺失。由于标本数太少，难以解释。对于上述可能的推测为携带*EGFR*基因突变的肿瘤细胞已被吉非替尼杀死或较好抑制，因此没有或极少携带*EGFR*基因突变肿瘤细胞残留在组织标本中；同样无或极少突变的DNA碎片释放入血。因此，测定血液中DNA突变有一定困难，北京协和医院2011年报道^[11]^，手术确诊的Ⅰ期、Ⅱ期肺癌患者，术前1天-2天匹配的血清基因突变率仅4.5%，而Ⅲ期、Ⅳ期突变率为35.7%。对长期生存患者匹配标本（酪氨酸激酶抑制剂治疗前后）的对比分析可能有助于验证该假设。本研究样本量较小，期待有大样本临床研究评价EGFR突变和生存的关系。

有报道表明，Ki-67蛋白表达可能与NSCLC患者预后相关，研究还显示其参与了肿瘤旁路、恶性肿瘤转化及疾病进展。既往研究报告Ki-67蛋白表达阳性率约30%，表达阳性者生存期较短。本研究3例肿瘤组织标本中2例表达阳性，吉非替尼治疗疗效持续时间均超过2, 000天。由于样本量受限，因此期待今后再行大样本量检测以评估Ki-67蛋白表达意义。
